# The role of enhanced recovery after surgery (ERAS) in promoting quality improvement and patient safety in pediatric urology

**DOI:** 10.3389/fruro.2023.1275276

**Published:** 2023-09-28

**Authors:** Darren Ha, Kelly T. Harris, Megan A. Brockel, Kyle O. Rove

**Affiliations:** ^1^ Pediatric Urology Research Enterprise, Department of Pediatric Urology, Children’s Hospital Colorado, Aurora, CO, United States; ^2^ Division of Urology, Department of Surgery, University of Colorado Denver Anschutz Medical Campus, Aurora, CO, United States; ^3^ Department of Pediatric Urology, Children’s Hospital Colorado, Aurora, CO, United States; ^4^ Department of Anesthesiology, Children’s Hospital Colorado, Aurora, CO, United States

**Keywords:** enhanced recovery after surgery, opioid reduction, length of stay, quality improvement, implementation science

## Abstract

Enhanced Recovery After Surgery (ERAS) is a set of evidence-based, multidisciplinary protocols that aim to improve the perioperative experience for patients by optimizing factors before, during, and after surgery. Originally developed for adult colorectal surgery, these protocols have expanded and been adopted into the pediatric surgical realm, including pediatric urology. Preoperative interventions are directed toward reducing physiologic and emotional stress prior to surgery, including preoperative education and decreased duration of fasting. Intraoperative interventions are designed to support physiologic homeostasis through maintenance of normothermia and euvolemia, use of regional anesthesia, and minimizing placement of drains. Postoperative interventions seek to reduce the physiologic burden of surgery and restore patients to their functional baseline through early oral intake, early mobilization, and opioid-sparing, multimodal analgesia. ERAS has demonstrated efficacy and safety across a wide variety of surgical subspecialties. In pediatric urology, ERAS has led to earlier return of bowel function, decreased opioid utilization, and shorter hospital length of stay, without an increase in complications compared to prior standard of care. ERAS can thus be seen as a system through which quality improvement (QI) initiatives can be designed and tailored to particular settings and patient populations. This review aims to summarize current data in pediatric urology regarding ERAS elements in the context of QI and patient safety. It will discuss the barriers and future directions of this field, including collaboration with implementation science to facilitate adoption of these protocolized measures more widely.

## 1 Introduction

Surgery places physiologic and emotional stress on patients and families. Enhanced Recovery After Surgery (ERAS) is a perioperative, multidisciplinary set of protocols aimed at reducing the physiologic burden of surgery (1). Pioneered by Danish surgeon, Dr. Henrik Kehlet in the late 1990s, the main tenets of ERAS include preoperative education, limiting preoperative fasting, nutritional support, use of minimally invasive approaches, judicious use of intraoperative opioids, minimization of indwelling tubes/catheters, early postoperative mobilization, and use of multimodal analgesia (1, 2). The first protocols were developed in 2001 for adult colorectal surgery and have since expanded to encompass urology, gynecology, and otolaryngology, among other surgical subspecialties (1, 3). Widespread adoption of ERAS in pediatrics was slow, owing in part to unique pediatric physiology, different nutritional demands, and limited ability of patients in this population to communicate (4). Elements of ERAS are currently used in pediatric surgical subspecialties such as colorectal surgery, otolaryngology, and urology (5–7). Within pediatric urology, ERAS pathways have been studied in children undergoing operations ranging from pyeloplasty to complex lower urinary tract reconstructions involving augmentation cystoplasty and/or urinary diversion (8, 9). Additional consideration has been given to children with comorbidities, such as spina bifida or ventriculoperitoneal shunts, to ensure that a greater proportion of patients can benefit from these interventions (10, 11). In the evolving landscape of quality improvement (QI) in healthcare, ERAS is a central component to value-based care and a valuable tool that balances safety, outcomes, and surgical experiences for patients and families. This review will focus on the evidence in support of the efficacy of ERAS in pediatric urology, particularly as it pertains to QI initiatives and advancement of patient safety.

## 2 Preoperative elements

### 2.1 Preoperative education

Engaging patients and families through preoperative education is the initial step in ERAS buy-in, regardless of surgical specialty or procedure type. Besides the duty to disclose relevant information to patients, effective preoperative education has been shown to reduce anxiety and improve the overall surgical experience (12, 13). Education should be age-appropriate and include active involvement of older children and adolescents, as appropriate, in order to promote engagement and set expectations (14). In a systematic review of ERAS in pediatric urology, increased preoperative counseling was one of the most common elements implemented, suggesting the importance of this step in the surgical journey (4). Our institution has established a multidisciplinary, virtual preoperative clinic visit involving surgeons, anesthesiologists, psychologists, and nurses to provide comprehensive education and describe the tailored role of ERAS principles in their specific surgery. This is an opportunity for the team to establish a foundational relationship with patients and families.

### 2.2 Setting parental expectations

Parental involvement is a salient component of ERAS in the pediatric population, as they are key stakeholders in the care of the patient. Engaging parents begins early with preoperative education to facilitate buy-in, which may augment patient participation and adherence to ERAS protocols, especially in older children (15). Setting and managing expectations about their roles in patients’ recoveries and emphasizing criteria for discharge based on postoperative milestones rather than length of stay have been cited as important components (16, 17). Education for parents must be robust and comprehensive as they take on the roles of pain control, refeeding, and monitoring for complications, particularly as earlier discharge is becoming the norm with ERAS (15). Specifically, education on pain management at home is vital. One study found that discharge instructions tailored to patients’ analgesic needs in the hospital and guidance on use of multimodal analgesia led to increased parental satisfaction and may have contributed to decreased opioid utilization at home (18). This point was echoed in a study utilizing a focus group to elicit parents’ perspectives for improvement in the surgical neonatal intensive care unit experience, and feedback was ultimately implemented into local ERAS protocols. Parents also noted that excessive paperwork with medical jargon was overwhelming, which prompted transition to readable informational handouts (17). The ERAS principle of a multidisciplinary team approach must necessarily involve parents as core members to facilitate investment, improve satisfaction, and advance patient safety.

### 2.3 Prehabilitation

The concept of prehabilitation refers to preoperative exercise, nutritional, and psychological support to bolster patients’ ability to tolerate stressors associated with surgery. The majority of work in prehabilitation thus far comes from adult surgical oncology, where nutritional optimization has been studied as a method to counteract cancer cachexia and/or chemotherapy side effects prior to surgical intervention. Suboptimal preoperative nutritional status and frailty contribute to perioperative complications and impaired wound healing, but nutritional supplementation has been demonstrated to improve outcomes (19). Data are promising insofar as they show decreased rates of postoperative infections and hospital length of stay when combined with ERAS principles. Immunonutrition, which involves fortifying nutrition with certain amino acids or fatty acids, is another adjuvant therapy thought to help modulate the host immune response after surgery, although current evidence is limited (20). Preoperative exercise programs have shown similar benefits in reducing postoperative complications through increased physical reserves. In specific cases, there is potential for patients who were previously considered poor surgical candidates due to deconditioning to be considered for surgery with use of these programs (21). Meanwhile, preoperative psychological support aims to encourage behavioral modifications (e.g., smoking cessation) and promote mental well-being through stress and anxiety reduction. While behavioral changes may not be as relevant in pediatrics, there is room to incorporate the latter into preoperative interventions. Currently, there is low quality evidence in support of psychological prehabilitation for patient outcomes (22). Notably, there is a paucity of data on prehabilitation in the pediatric population. While frailty may not be as common in children, those with reduced functional capacity prior to surgery may benefit from additional therapies to build reserves for optimized recovery. Furthermore, insulin resistance and inflammation have been implicated as drivers of postoperative metabolic derangements, so children with proinflammatory conditions may be another target population for these programs (23). Evaluating the use of preoperative psychological support for parents and caretakers may also yield important findings with implications for additional preoperative counseling and services and building this into pediatric ERAS pathways will likely improve the surgical experience for patients and families.

### 2.4 Preoperative fasting and carbohydrate loading

Extended fasting prior to general anesthesia was rooted in the idea that it decreased gastric volume and acidity and reduced the risk of regurgitation and aspiration of gastric contents during surgery (24). However, a Cochrane review found that children permitted fluids up to two hours prior to surgery did not have higher gastric volumes or lower gastric pH compared to those who fasted. Furthermore, these children were less hungry and thirsty and more comfortable. Among the analysis of more than 2500 children, there was only one reported case of aspiration (25). A shorter duration of fasting has been widely adopted in the pediatric urology realm for nearly all surgical procedures. The safety of this approach has been consistently borne out of data demonstrating no difference in complications before and after implementation (4, 9). Additionally, prolonged fasting has been associated with a catabolic state and insulin resistance (26). The aim of carbohydrate loading prior to surgery aims to reduce these physiologic derangements and lower the stress response (27). Data from the adult perioperative literature have shown that drinking carbohydrate-rich clear liquids up until two hours before surgery resulted in less hunger and thirst compared to those who fasted or drank non-caloric clear liquids (28). Meanwhile, evidence in pediatrics suggests that a clear liquid carbohydrate drink may promote a more stable perioperative metabolic state, particularly for longer operations (29). Based on existing data, a group of pediatric urologists and anesthesiologists have incorporated a preoperative clear liquid complex carbohydrate load into their revised ERAS protocols for lower urinary tract reconstructions (30).

### 2.5 Elimination of mechanical bowel preparation

Preoperative mechanical bowel preparation was popularized in the 1970s due to purported benefits of decreasing complications and infections associated with surgeries involving the intestines (31). Efforts to challenge this dogma have been incremental. An early retrospective study comparing outcomes and complications in children undergoing augmentation cystoplasty with versus without mechanical bowel preparation found that those who were spared from bowel preparation had shorter median length of stay (LOS), earlier time to postoperative oral intake, and similar rates of infections and anastomotic leaks (32). Another study looked specifically at 30-day postoperative complications in children undergoing augmentation cystoplasty without bowel preparation. They reported no intraoperative complications and an overall 30-day postoperative complication rate of 9.87%, which was equivalent to existing rates in the literature (33).

There has been concern related to eliminating mechanical bowel preparation in the subset of patients with spina bifida with ventriculoperitoneal (VP) shunts due to the risk of shunt infections, which can be devastating (32). A study of children with VP shunts undergoing augmentation cystoplasty using bowel found no difference in the rate of shunt infections between those who did and did not undergo bowel preparation (34). Similarly, there were no VP shunt infections following introduction of an ERAS protocol for pediatric urinary tract reconstruction that included elimination of a dedicated, preoperative bowel preparation and maintenance of routine outpatient bowel regimens in those with concomitant neurogenic bowel (10). This particular example illustrates the consideration in outcomes among different patient populations when evaluating whether intended effects of ERAS elements are equally distributed. Overall, foregoing this step has been a welcome addition to ERAS protocols for many institutions due to improved perioperative experiences for patients and reductions in costs associated with decreased LOS (9, 30, 31).

## 3 Intraoperative elements

### 3.1 Maintenance of normothermia

Anesthetic-induced inhibition of thermoregulation, in addition to exposure of body surface area to the cooler operating room environment, can lead to perioperative hypothermia, defined as a body temperature of less than 36.0°C during the perioperative period (35). Even a mean decrease in body temperature of 1.5°C has been associated with increased likelihood of requiring a blood transfusion and developing a postoperative infection, with subsequent increased costs of care related to managing these complications (36). A Cochrane review also found a similar benefit of perioperative normothermia in mitigating surgical site infections and complications in patients undergoing abdominal surgery (37). Since younger patients are more susceptible to anesthetic-induced thermodysregulation, maintenance of appropriate body temperature during surgery should be a priority for safety and cost-conscious care (35). The Pediatric Urology Recovery After Surgery Endeavor (PURSUE) multicenter study has incorporated maintenance of normothermia—defined as body temperature of 36°C–38°C during skin-to-skin time—into their revised ERAS protocol for lower urinary tract reconstruction (30).

### 3.2 Maintenance of euvolemia

Fluid management has been a mainstay of ERAS protocols since their inception. Hypovolemia predisposes patients to renal injury, while hypervolemia increases the risk for cardiorespiratory complications, impaired wound healing, and delayed recovery (38). One study found that for each additional liter of fluid given, the risk of postoperative symptoms delaying recovery increased by 16% while the risk of postoperative complications increased by 32% (39). Within pediatric urology, special attention is paid to renal function as many urologic patients have or are at risk of renal injury as a function of their disease processes. Thus, achieving intraoperative euvolemia with the goal of maintaining adequate renal perfusion is of the utmost importance (40). This tenet is reflected in the ubiquitous adoption of minimizing excessive intraoperative fluids in both pediatric colorectal surgery and pediatric urology (4, 7, 10, 41, 42).

Prior standard of care involved administering a fluid bolus to account for presumed fluid deficits in the context of prolonged fasting, followed by intraoperative maintenance fluids based on the Holliday and Segar formula (43). Given minimization of prolonged fasting and the risks associated with hypervolemia, as discussed, goal-directed fluid resuscitation is now commonplace. However, assessing for euvolemia has been imperfect. Methods such as the pleth variability index (PVI), stroke volume index, transesophageal echocardiography, and esophageal doppler have been studied as proxies for hemodynamic variables and fluid responsiveness during surgery (44). While some studies show the predictive value of PVI in guiding goal-directed fluid therapy, research in PVI has generally lacked standardization in types of fluid given, volume administered, and definition of fluid responsiveness, thus limiting the conclusions that can be drawn (45). Currently, no single method has been shown to be superior. Nonetheless, goal-directed fluid therapy has been shown to significantly decrease the volume of intraoperative fluids administered and reduce surgical morbidity (43, 46).

### 3.3 Regional and multimodal analgesia

The opioid epidemic has galvanized the medical community into reducing reliance on opioids for pain control. Operative pain management may be the context of children’s first exposure to opioids. This patient population may be more vulnerable to misuse compared to adults due to alterations in the reward and habit centers in the brain, with some data reporting that approximately 5% of opioid-naïve adolescents and young adults continue to fill opioid prescriptions more than 90 days after surgery (47). With evidence demonstrating similar efficacy of NSAIDs to opioids in managing postoperative pain in children, the transition to regional and multimodal analgesia starting in the operating room has become standard for many pediatric surgeries (4, 41, 48, 49).

A prospective case-control study found that children undergoing urologic reconstructive surgery with adherence to ERAS protocols were more likely to receive intraoperative dexmedetomidine, acetaminophen, and NSAIDs, with a resultant decrease in intra- and postoperative opioids compared to historical controls (50). Another study sought to characterize factors associated with same-day discharge for pediatric pyeloplasty between 2008–2020. The authors identified a trend of greater utilization of ketorolac and regional blocks and a decrease in opioid use throughout the years, which was reflected in a shorter LOS (8). Among children with spina bifida undergoing complex lower urinary tract reconstruction, use of regional analgesia resulted in a 70% intraoperative and 78% postoperative, in-hospital reduction in opioid use without higher pain scores. The authors note that use of regional analgesia as part of ERAS protocols confers the benefits of opioid minimization to this subset of patients where neurologic function and sensation may be altered (11).

The burden of the opioid epidemic has increased costs in sectors spanning from healthcare, substance use treatment, criminal justice, and the labor market (51). Viewed through the lens of QI and patient safety, minimizing perioperative opioid use has the implication to mitigate immediate adverse health outcomes as well as far-reaching consequences associated with future opportunity costs for patients.

### 3.4 Minimizing placement of tubes and drains

Placement of tubes and drains at the conclusion of surgery has long been standard practice in order to facilitate surgical site drainage and prevent fluid collections and infections. However, this intervention contributes to postoperative pain and discomfort, limits mobility, and negatively impacts quality of life for patients (1). Evidence from the adult literature does not show a benefit for prophylactic drain placement in a myriad of surgeries (52). Similarly, drain placement has not been associated with improved outcomes in select pediatric surgeries, including upper urinary tract reconstruction (53–55). Numerous studies in pediatric urology have successfully incorporated this component into their enhanced recovery protocols without an increase in adverse events, further supporting the safety of this approach (8, 10, 50, 56). Current evidence seems poised to obviate operative drain placement in many pediatric urologic procedures.

## 4 Postoperative elements

### 4.1 Early oral intake

Patients were routinely *nil per os* (NPO) after surgery to mitigate postoperative nausea and vomiting. In the setting of surgeries that involved the gastrointestinal tract, it was purported that avoiding immediate oral intake would protect anastomoses from the stress of feeding (57). Certain pediatric urologic procedures, such as enterocystoplasty, involve the use of bowel segments to reconstruct the urinary tract. Thus, by extension, children undergoing these types of procedures should also be kept NPO after surgery to preserve bowel integrity. However, a systematic review of eleven randomized controlled trials comparing postoperative NPO versus early oral refeeding within 24 hours after colorectal surgery found no clear benefit to restricting oral intake. While early refeeding did lead to an increased risk of vomiting, it was associated with decreased risk of anastomotic dehiscence and infections of any type, ultimately leading to shorter LOS (57). Early postoperative refeeding has been implemented and studied in numerous pediatric surgical subspecialties and been found to be a welcome addition leading to earlier return of bowel function, an important criterion for hospital discharge (7, 41, 58). In adult urology, early oral intake spared patients undergoing open radical cystectomy from five additional days of fasting and resulted in earlier time to bowel movement (3.64 versus 6 days), without a difference in 90-day complication rates (3). This outcome was reproduced in pediatric urology as well (4). Even amongst children undergoing bladder augmentation and/or urinary diversion, early refeeding has been associated with earlier return of bowel function (9, 10).

### 4.2 Minimization of opioid use

Efforts to minimize opioid use begin intraoperatively and continue into the postoperative period. The emphasis on opioid stewardship is particularly important after surgery because of discrepancies in provider prescribing patterns (59, 60). Even as recently as 2017, some children received up to 24–26 days’ supplies of opioids following routine hernia repairs and tonsillectomies (61). Within pediatric urology, one study found that 99% of children were prescribed opioids after surgery (62). One possible driver of this pattern of excessive opioids is concern regarding potential pain crises (63). However, current evidence suggests ERAS patients have well-controlled pain, both in-hospital and after discharge. PACU pain scores were significantly lower in ERAS cohorts who were managed with adjunct non-opioid medications such as acetaminophen and NSAIDs (41, 50). Meanwhile, 80% of children had non-significant levels of pain by postoperative day one (64). In those who were prescribed opioids, most used five doses or less, typically within the first three days after surgery (65). Even a majority of pediatric urologists surveyed did not believe that patients used all opioid doses prescribed (66). Opioid utilization patterns corroborate this belief, with up to 62% of patients reporting unused doses two weeks postoperatively (62). Thus, the duration of recovery after surgery represents a critical period for intervention in reducing opioid use. Reassuringly, studies in the pediatric population have consistently demonstrated successful reduction in postoperative opioid use upon adoption of ERAS protocols (4, 7, 10, 41, 50).

## 5 Evidence in support of quality improvement and patient safety

T/he data presented thus far validate the success of ERAS in isolated measures. Whether that is improvement in quality of life by reducing preoperative fasting and eliminating bowel preparation, reducing the risk of infection and hypervolemia by maintaining intraoperative normothermia and euvolemia, or expediting milestones to safe discharge with early postoperative refeeding—current evidence supports the transition away from historical practice to these updated policies. However, assessing the efficacy of ERAS as a whole also involves examining the overall impact of these protocols on global outcomes and complications. **Table 1** summarizes interventions and outcomes in select pediatric urology studies.

**Table 1:** Summary of ERAS interventions and outcones in pediatric urology.

Reference	Surgeries performed	Number of patients (ERAS/total)	Patient age range	Intervention(s)	Outcomes measured
Gundeti et al (2006) (18)	Bladder augmentation using small bowel	22/46	3.3-18 years ERAS 4-20 years non- ERAS	No preoperative mechanical bowel preparation; normal preoperative diet	Time to oral fluids (24 hrs ERAS versus 48 hrs non-ERAS) LOS (4 days ERAS versus 5 days non- ERAS) 2 postoperative urinary tract infections ERAS versus 1 non-ERAS No anastomotic leakage or bowel obstruction in either group
Victor et al (2012) (19)	Bladder augmentation using bowel	158	2.1-22.7 years	No preoperative mechanical bowel preparation	Time to oral fluids (mean 94.77 hrs) LOS (mean 9.48 days) Postoperative urinary fistula (2.4%) Postoperative wound infection (1.85%) Need for reoperation (3.16%)
Haid et al (2018) (13)	Bladder augmentation and urinary diversion using small bowel	15/30	9.27 ± 1.14 years ERAS 10.93 + 1.24 years non-ERAS (mean ± SD)	Pre-surgical counseling and education; no prolonged preoperative fasting; fluid and carbohydrate loading; no preoperative mechanical bowel preparation	Bowel-related complications (ns) Time to flatus (2.8 ERAS versus 4.7 days non-ERAS, p=0.002) Time to stool (3.33 ERAS versus 5.53 days non-ERAS, p=0.002) LOS (11.93 ERAS versus 19.87 days non- ERAS, p<0.001)
Rove et al (2018) (27)	Lower urinary tract reconstruction with bowel anastomosis	13/39	9.9 years (9.1-11.0) ERAS 10.4 years (8.0- 12.4) non-ERAS [median (IQR)]	Preoperative counseling and carbohydrate load; use of regional anesthesia; avoidance of excess drains; maintenance of euvolemia; use of opioid- sparing analgesia; early enteral feeding; early mobilization	Time to clear liquids (0 days ERAS versus 1 day non-ERAS, p<0.001) Time to regular diet (1 day ERAS versus 4 days non-ERAS, p=0.002) Return of bowel function (2 days ERAS versus 4 days non-ERAS, p=0.002) LOS (5 days ERAS versus 6 days non- ERAS, ns) Readmission within 30 days (1 ERAS versus 7 non-ERAS, ns) Reoperation within 90 days (1 ERAS versus 8 non-ERAS, ns) ED visits within 90 days (7 ERAS versus 17 non-ERAS, ns) Total complications within 90 days (17 ERAS versus 56 non-ERAS, p=0.04)
Chan et al (2021) (39)	Lower urinary tract reconstruction	20/40	11.3 years (4.1- 21.4) ERAS 11.4 years (7.7- 25.1) non-ERAS [median (IQR)]	Preoperative counseling; no prolonged preoperative fasting; no preoperative mechanical bowel preparation; use of regional anesthesia; maintenance of normothermia and euvolemia; opioid-sparing analgesia; early enteral feeding; early mobilization	LOS (4 days ERAS versus 9 days non- ERAS, p<0.05 Readmission within 30 days (6 ERAS versus 4 non-ERAS, ns) Reoperation within 30 days (3 ERAS versus 6 non-ERAS, ns) Total complications within 30 days (19 ERAS versus 19 non-ERAS, ns)

ERAS, enhanced recovery after surgery; LOS, length of stay; ED, emergency department.

### 5.1 Length of stay and patient satisfaction

Perioperative care has been proposed as a significant driver of surgical outcomes, not the surgery itself (1). It has been suggested that mortality may not be an appropriate indicator of success in the pediatric ERAS realm (4). Hence, length of stay has, and continues to be, an important objective measure of the cumulative impact of ERAS elements, as it reflects an improvement in systems of care. Time and time again, ERAS has resulted in reductions in hospital LOS for children across different surgical specialties that are both statistically and clinically significant (7, 9, 41, 42, 58). One study in children undergoing urologic reconstruction noted that the greater the number of ERAS elements implemented, the shorter the LOS (10). This suggests an additive effect of individual elements in contributing to outcomes. It has been observed that there are fewer ERAS elements carried out in pediatrics compared to adults, pointing to potential room for further improvement in reduction of LOS (58). Another meaningful outcome is patient and family satisfaction. While inherently subjective, this measure is arguably just as important since data on patient experience are often gathered during QI initiatives in order to optimize subsequent encounters. Early studies in pediatric ERAS have described high levels of patient and family satisfaction as a result of participation (4, 58). Additional work is necessary to fully characterize the surgical experience and gather more granular data on areas for improvement.

### 5.2 Complications

Evaluating ERAS through the lens of patient safety involves scrutinizing complication rates compared to prior standard of care. Studies in pediatric colorectal and minimally invasive surgeries reported similar postoperative complication rates before and after adoption of ERAS (7, 41, 42, 67). Results from pediatric urology also demonstrated complication rates that were not worse compared to pre-ERAS cohorts (33). In fact, ERAS has been associated with fewer bowel-associated and total complications, with similar rates of postoperative emergency department visits, readmissions, and reoperations (4, 9, 10, 56). These robust data support ERAS as a tool to promote QI and patient safety.

### 5.3 Costs

Maximizing quality of care while minimizing cost is a considerable motivation for QI initiatives within the realm of surgery and it has been suggested that ERAS protocols play a part through reductions in hospital LOS and patient morbidity (68). While it can be difficult to quantify exact cost savings, limited studies have demonstrated that ERAS implementation is associated with cost savings for patients (1). A systematic review of costs in the adult surgical literature showed reduced in-hospital costs for those undergoing esophageal, gastric, pancreatic, colorectal, bariatric, vascular, and gynecologic surgeries with ERAS protocols (68). There is a paucity of data for pediatric surgeries but a decrease in hospital costs has been reported for children undergoing colorectal surgery (4, 7). Two other studies found that costs were not higher after implementation of ERAS for pediatric surgeries (69, 70). Meanwhile, a QI initiative at a pediatric ambulatory surgical center noted that their cost reduction was driven by substitution of intravenous acetaminophen with ketorolac (71). While additional cost analyses are necessary, particularly in pediatric urology, it should be acknowledged that in-hospital economic evaluations cannot adequately account for indirect societal costs associated with factors such as children’s need for postoperative, in-home support leading to potential reductions in caregiver wages (68). Thus, future studies that incorporate these indirect costs will illustrate a more comprehensive picture of the true cost impacts from ERAS. Nonetheless, current evidence indicates that ERAS provides good value-based care (1).

## 6 Barriers and future directions

The extent of published evidence demonstrates the safety and effectiveness of ERAS protocols for the pediatric population, however, adoption in pediatric urology remains slow (72). A survey of pediatric urologists found that 38% of respondents lacked familiarity with enhanced recovery pathways. Among those with familiarity, lack of consensus with other pediatric urologists (62%), lack of administrative support (56%), difficulty initiating and maintaining pathways (38%), and lack of anesthesia support (31%) were among the most commonly cited barriers to implementation and/or standardization (73). Lack of administrative support was echoed as a barrier in a study of pediatric surgeons (74). A QI initiative to decrease perioperative opioid use in children also noted inadequate care team buy-in as a limiting factor to optimal implementation (71). Meanwhile, 90% of pediatric urology survey respondents were willing to implement some elements of ERAS into their practice, suggesting a willingness, or even demand, for such pathways (73).

While the obstacles to implementing such an extensive protocol may seem numerous, they must be weighed against the benefits for patient care and safety. A QI initiative to implement enhanced recovery pathways in 20 children undergoing bladder reconstruction demonstrates the feasibility of such an endeavor and highlights the assets of this approach (56). Designated champions communicated changes to their teams and met regularly for audits. Time from planning to first implementation was seven months and protocol elements led to a significant decrease in LOS (4 versus 9 days, p<0.05) without an increase in 30-day complications. A median of 16 out of 24 ERAS elements (67%) were implemented, which was below their goal of 80% adherence, but it still resulted in significant improvements in their outcome measures.

Pioneers in ERAS claim that 70–80% adherence to ERAS elements is necessary to improve outcomes (1). The current literature reveals that additional work is necessary to achieve this standard, with adherence rates ranging from 45–62%; meanwhile only 16% of cohorts were able to achieve at least 75% adherence in one study (42, 73, 74). However, the improvements in patient outcomes despite suboptimal adherence to all elements lends support to the effectiveness of these protocols. Furthermore, while some studies cite low ERAS implementation rates among pediatric surgeons, many currently implement individual elements in their practice but do not label it as ERAS, suggesting a degree of underreporting (41, 73, 74).

The future of ERAS in pediatric urology will likely involve additions or alterations in the elements implemented. For example, recent data from the adult surgical literature have shown that patients allowed to drink clear liquids up until arrival in the operating room did not have worse outcomes, opening up the potential for future adoption in the pediatric domain to further decrease discomfort associated with fasting (75). In fact, the European Society of Anesthesiology and Intensive Care recently put forth pediatric guidelines permitting intake of clear fluids up until one hour before surgery (76). Another major avenue for future improvement involves identifying and addressing barriers to adoption of ERAS among a larger proportion of pediatric urologists. This will require collaboration with experts in implementation science, a field that studies and develops strategies to promote adoption of evidence-based interventions into clinical practice, with a focus on how to optimize delivery to achieve the most impact (77). Replicating successful implementation from one setting to another can be difficult due to various contextual factors, so asking *why* an initiative was successfully implemented is just as important as asking *how* an initiative produces improved outcomes (78).

Popular frameworks within implementation science include Consolidated Framework for Implementation Research (CFIR); Exploration, Preparation, Implementation, Sustainment (EPIS); and Normalization Process Theory (NPT) (**Table 2**) (77). NPT—which focuses on behaviors and attitudes that allow an intervention to become incorporated into routine practice and no longer seen as an intervention—has been utilized in the setting of implementing several ERAS protocols (79). Lam and colleagues implemented an ERAS protocol for neonatal intestinal resection and outlined recommendations for successful adoption in alternate settings as viewed through an NPT lens: identifying champions from different teams, gathering multidisciplinary stakeholder buy-in, tailoring interventions to local needs, eliciting patient and family engagement, and performing audits for process improvements (80). Another study applied the NPT framework to understand factors that promoted ERAS implementation in adult thoracic, colorectal, and head and neck surgeries. The authors found that differentiating ERAS from prior standard of care and positive beliefs in the value of ERAS, both individually and as a team, facilitated successful implementation (81). The CFIR framework has also been used to identify effective team handoffs, robust post-discharge support, and promotion of patients’ self-efficacy in recovery as factors associated with successful implementation of ERAS in adult orthopedic surgery (82).

**Table 2:** Summary of core components of 3 common implementation science frameworks.

Consolidated Framework for Implementation Research (CFIR)	Exploration, Preparation, Implementation, Sustainment (EPIS)	Normalization Process Theory (NPT)
Structured schema for implementing system-wide interventions through assessment of five domains: 1) intervention (e.g., evidence and cost), 2) inner setting (e.g., clinic or hospital context for intervention), 3) outer setting (e.g., economic and societal factors), 4) individuals involved, and 5) processes (e.g., planning, execution, and audits of implementation).	Consideration of contexts and variables that influence successful implementation of an intervention at each of the four phases: 1) exploration (e.g., stakeholders identify a need and intervention is selected based on evidence-based research), 2) preparation (e.g., assess local context and adapt as necessary), 3) implementation (e.g., carry out intervention via trainings, feedback, etc.), and 4) sustainment (e.g., promote continued use of intervention through audits and evaluations).	Examination of behaviors and attitudes that promote the normalization of an intervention such that it is routine practice and no longer seen as an intervention. Components include: coherence (e.g., making sense of an intervention), cognitive engagement (e.g., participation in intervention), collective action (e.g., work put in to implement an intervention), and reflexive monitoring (e.g., evaluating costs and benefits of intervention).

**Figure 1:**
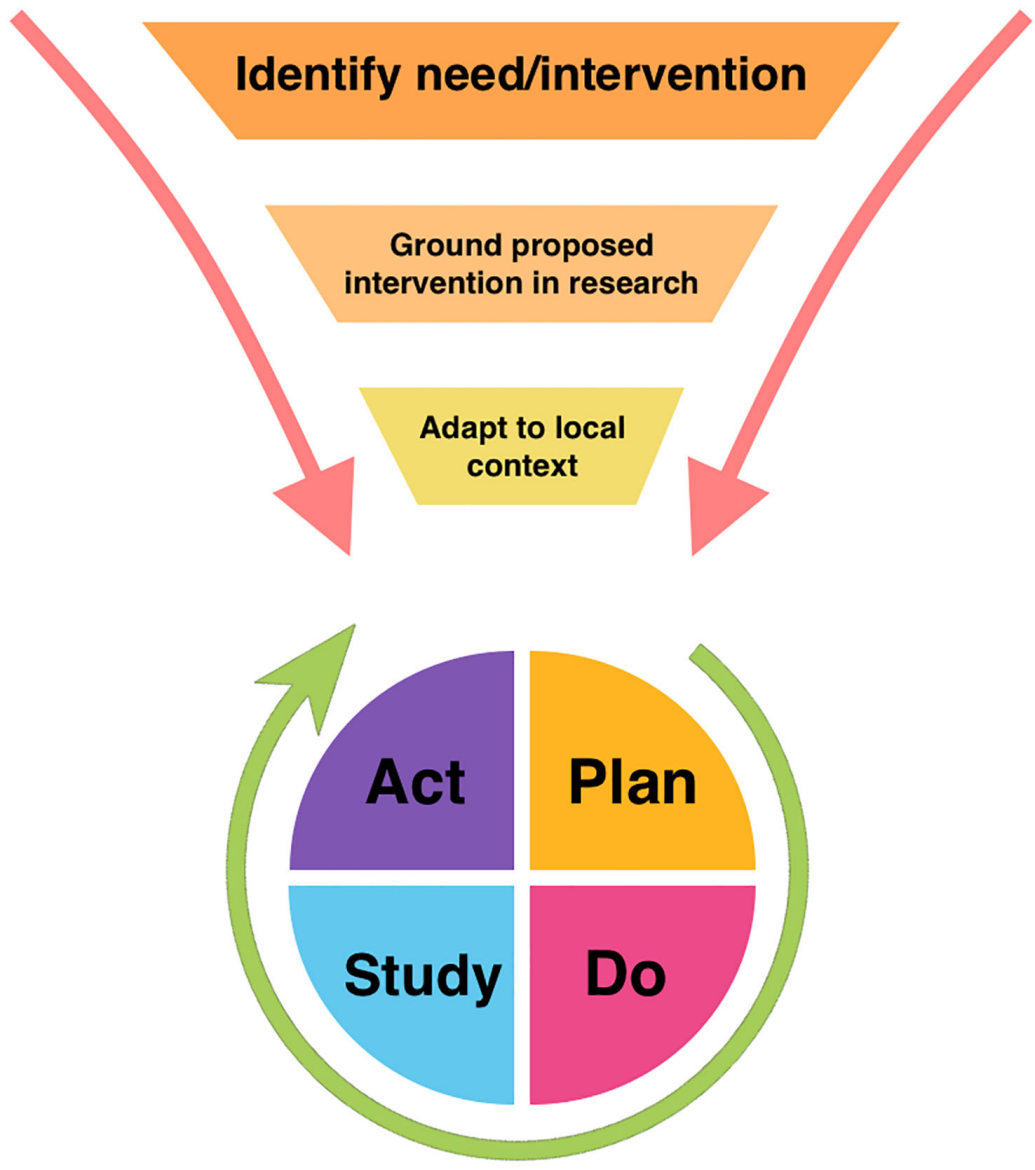
Model of relationship between broad implementation science principles and Plan-Do-Study-Act quality improvement framework.

As ERAS continues to gain traction in pediatric urology, drawing from work in implementation science will be an important component to overcome barriers to successful expansion. **Figure 1** illustrates how general implementation science principles can be harnessed to facilitate tailored adoption of an intervention, such as ERAS, which can then be studied and revised locally using QI frameworks.

## 7 Conclusions

Surgery can be a considerable undertaking for patients and families. ERAS protocols were developed to address and optimize the patient experience at every step of the surgical pathway. Studies repeatedly illustrate the safety and efficacy of ERAS in decreasing patient discomfort, perioperative opioid use, and hospital length of stay without increased complications compared to prior standard of care. The iterative process of implementation and audits demonstrates the role of ERAS in driving QI initiatives and promoting patient safety. This is a rapidly growing sector of research, particularly within pediatric urology, as providers strive to improve process measures through consideration and adoption of additional elements. As the field of pediatric urology ERAS continues to advance, the paradigm is shifting from the sole focus on its merits as a tool to optimize patient outcomes and safety to one that includes addressing barriers and facilitators to widespread implementation of these evidence-based interventions.

## Author contributions

DH: Writing – original draft, Writing – review & editing. KH: Writing – review & editing. MB: Writing – review & editing. KR: Writing – review & editing.
